# Bilateral Adrenal Hemorrhage in Pregnancy: A Clinical Report of a Rare Case

**DOI:** 10.7759/cureus.82453

**Published:** 2025-04-17

**Authors:** Gopi Patel, Risham Javaid, Grace Pena, Mohammad Al-Dmour, Raana Zafar, Sahar Farhan, Isma Jamshaid, Umar Farooq

**Affiliations:** 1 Department of Internal Medicine, Jamaica Hospital Medical Center, Queens, USA; 2 Department of Internal Medicine, Flushing Hospital Medical Center, Queens, USA

**Keywords:** adrenal insufficiency, bilateral adrenal hemorrhage, imaging in pregnancy, medical management, pregnancy

## Abstract

Adrenal hemorrhage in pregnancy, particularly of spontaneous origin, is an exceedingly rare phenomenon. We present the case of a 19-year-old primigravida, at 38 weeks gestation, afflicted by bilateral spontaneous adrenal hemorrhage. Initial presentation with severe abdominal pain posed diagnostic conundrums, necessitating a meticulous and multidisciplinary approach. Radiological evaluations revealed bilateral adrenal gland thickening, raising the suspicion of adrenal hemorrhage. The ensuing management included blood transfusion and steroid therapy, pivotal in stabilizing the patient's precarious clinical state. This case underscores the imperative of interdisciplinary collaboration and expeditious interventions in navigating medical complications during pregnancy as well as the importance of keeping in mind differential diagnoses such as adrenal insufficiency.

## Introduction

Adrenal hemorrhage is an uncommon occurrence in pregnancy. The most common causes of adrenal hemorrhage in pregnancy include blunt trauma, primary adrenal tumors, or metastatic tumors [1]. Spontaneous adrenal hemorrhage (SAH) is an uncommon condition with an estimated prevalence of 0.03-1.8% [2]. Symptoms on presentation can range from abdomino-pelvic pain and adrenal insufficiency (weakness, dizziness, nausea, and diarrhea) to frank shock secondary to adrenal crisis [3]. SAH primarily occurs unilaterally, with most cases reported to involve the right adrenal gland [4]. Bilateral involvement is extremely rare and can have marked clinical consequences for both the mother and fetus unless diagnosed and managed promptly. In this report, we present a case of bilateral SAH in a healthy pregnant young woman.

## Case presentation

A 19-year-old female at 38 weeks and four days of pregnancy presented with severe upper and lower abdominal pain, along with discomfort in her back and ribcage. The pain initially began as right upper back pain and later spread to both upper quadrants. She initially sought care at another hospital and received morphine, but the pain persisted. She also experienced loss of appetite and vomiting and could only tolerate clear liquids. Over-the-counter medications such as acetaminophen and diphenhydramine did not provide significant relief. The patient proceeded to have cramps and sharp pain, which matched contractions on fetal monitoring.

Physical examination

Upon examination, the patient was visibly distressed. While vital signs were normal, her blood pressure was slightly low. Her heart and lungs were normal, and no unusual masses were detected during palpation. Tenderness was present in the right upper abdomen.

Imaging findings

A sonogram of the upper right abdomen revealed a normal liver without signs of gallstones or inflammation. Mild right kidney swelling was observed, possibly due to pregnancy. Initially, the imaging could not pinpoint the cause of the severe abdominal pain.

**Figure 1:**
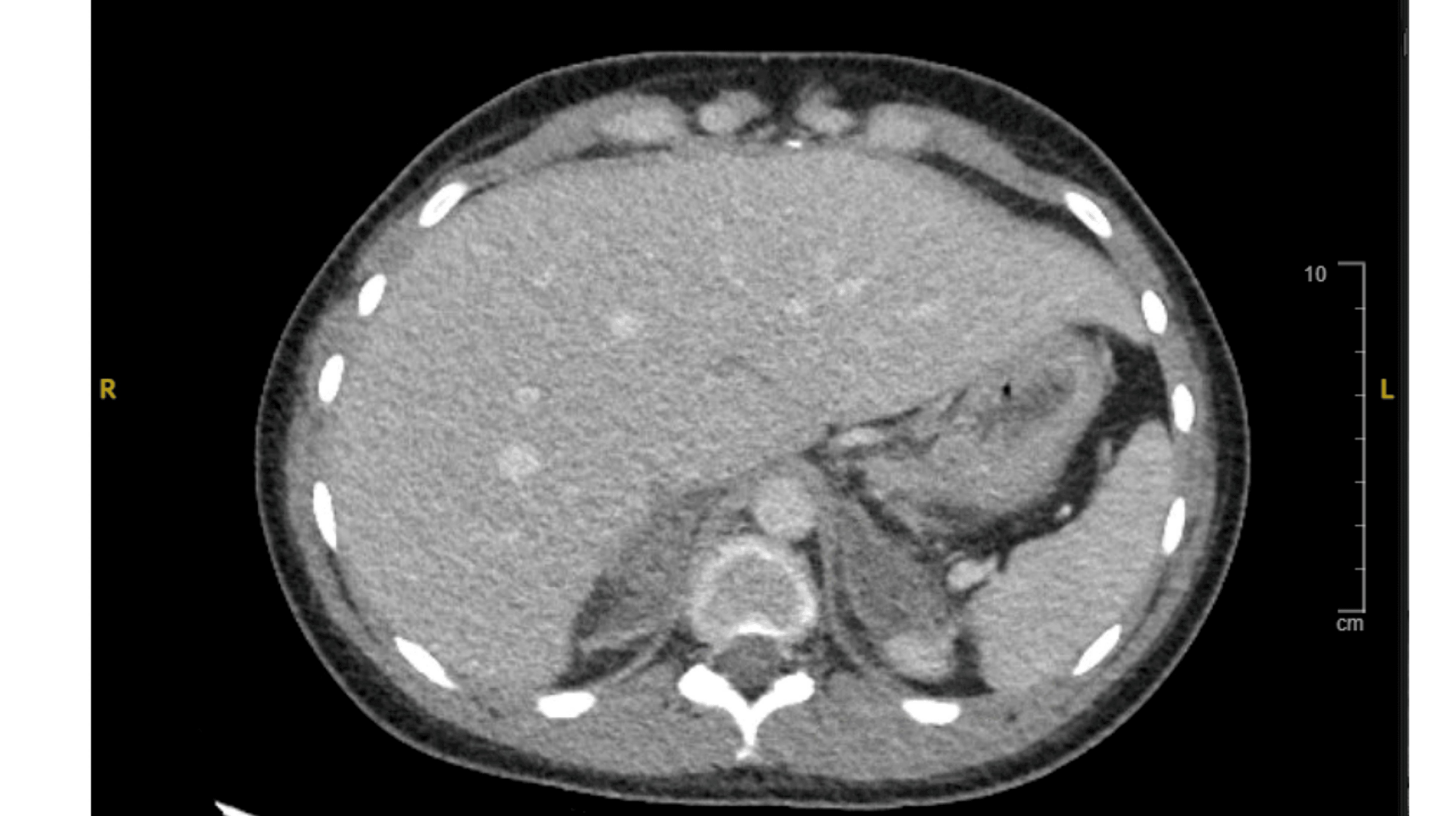
CT of the abdomen with and without contrast. There is marked adrenal gland thickening. Primary diagnostic consideration is bilateral adenomatous hyperplasia. Whether there is adrenal microhemorrhage is difficult to exclude based on the CT.

**Figure 2:**
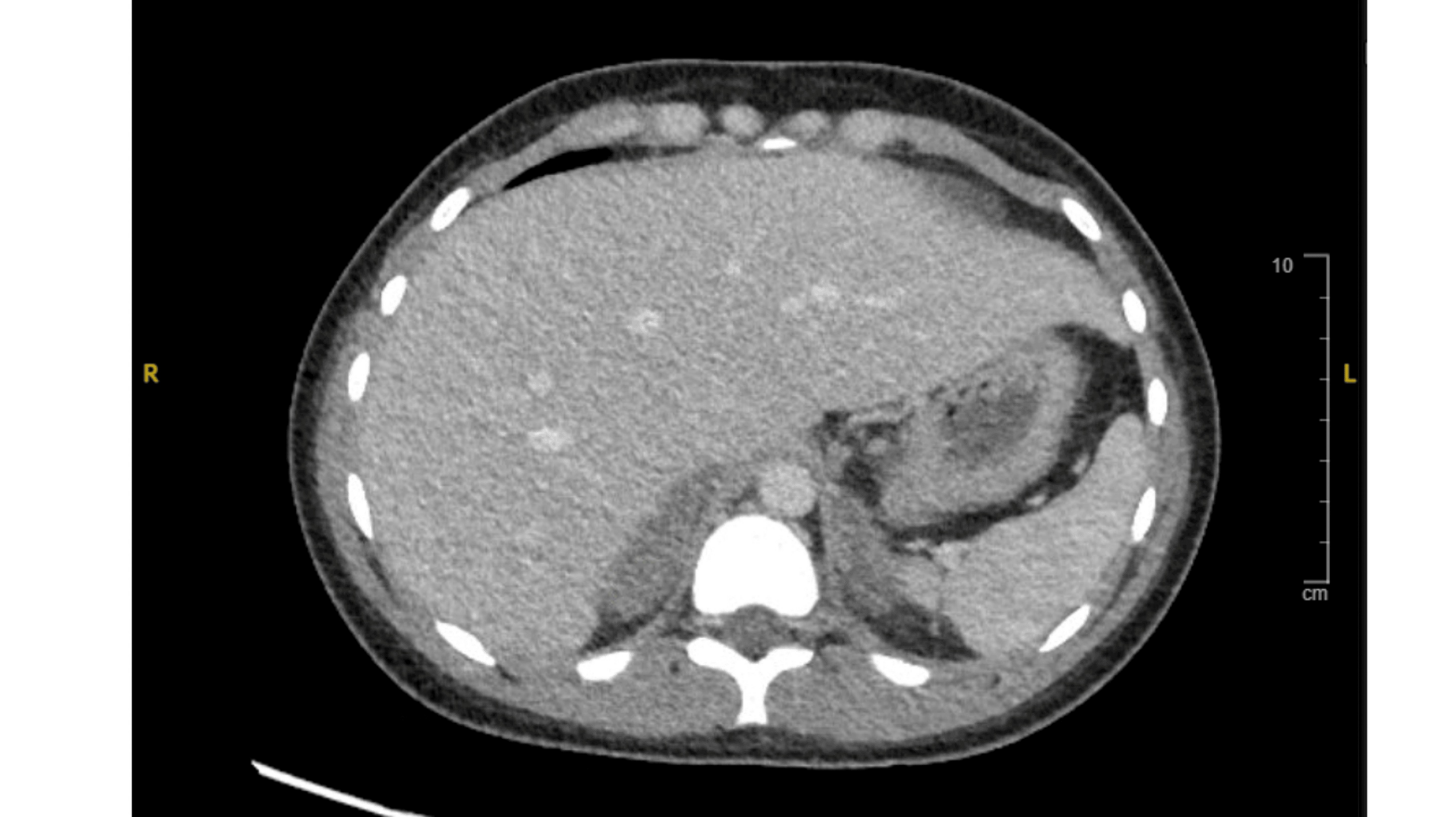
CT of the abdomen with and without contrast. There is marked bilateral adrenal gland thickening.

A complete abdominal ultrasound revealed a small amount of perihepatic ascites and mild right hydronephrosis, which could be secondary to the patient's pregnancy status. Though lungs were initially clear, new mildly prominent bibasilar markings appeared on X-ray on day 6. A kidney, ureter, and bladder (KUB) X-ray showed nonspecific bowel pattern and intrauterine pregnancy. CT scans revealed marked bilateral adrenal gland thickening without discrete masses, possibly indicating bilateral adrenal hemorrhage and/or adenomatous hyperplasia (Figure 1 and Figure 2).

Clinical progression and treatment approach

Considering the intensity of the distress and suspicion of intestinal blockage, an MRI of the abdomen was suggested for further examination. However, the patient could not undergo the MRI of the abdomen due to her being in significant pain. An MRI was attempted, but when she reached the MRI machine, she became anxious and refused to undergo the MRI. The patient initially was treated with conservative management, which included nasogastric tube insertion and administering a Fleet enema to facilitate bowel movement.

The patient's pain was addressed using fentanyl (50 mcg) every three to four hours as needed. A thorough review of her medical history revealed a previous incident of alcohol-related confusion and a past episode of upper right abdominal pain diagnosed as acute gastritis. The patient had also occasionally taken evening primrose pills during pregnancy. The obstetrician suggested using oxytocin to enhance labor progress due to concerns arising from fetal monitoring.

Post-delivery complications

**Figure 3:**
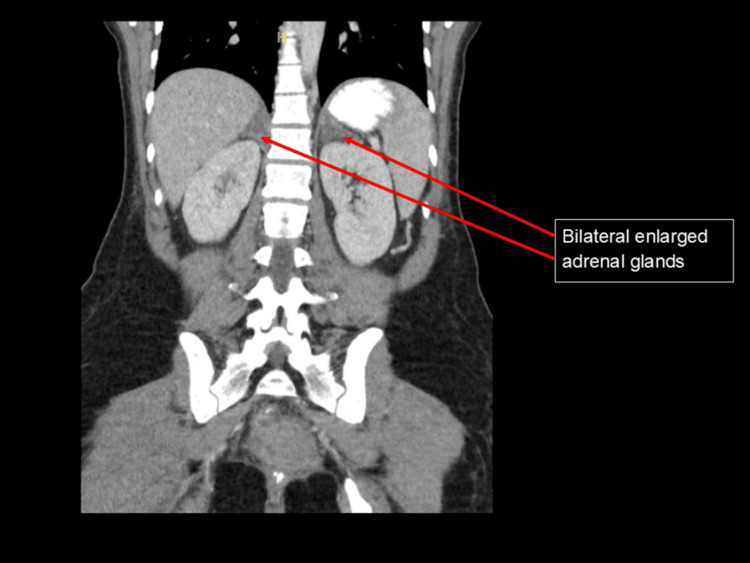
CT of the abdomen with and without contrast. Coronal view showing marked bilateral adrenal gland thickening.

After delivering a healthy baby girl, the patient faced acute anemia from blood loss, causing her hemoglobin levels to drop to 6.3 g/dL. She received a blood transfusion and started taking iron, vitamin C, and stool softeners. A fever with elevated white blood cell count emerged and was managed with antibiotics (ampicillin and gentamicin). On the second postpartum day, her condition worsened, leading to a transfer to the medical intensive care unit (MICU) due to suspected adrenal gland bleeding. An abdominal CT scan indicated symmetrically enlarged and varied adrenal glands, raising concerns of adrenal insufficiency (Figure 3).

Hydrocortisone (50 mg every six hours) was initiated, and the blood transfusion stabilized her hemoglobin levels. Further tests, including adrenocorticotropic hormone (ACTH) stimulation tests, confirmed adrenal insufficiency. Her cortisol response was diminished, prompting the start of prednisone (5 mg daily). She was discharged with follow-up plans and recommendations for endocrinology evaluation as an outpatient.

## Discussion

This case demonstrates a multifaceted medical scenario involving a pregnant patient with severe abdominal pain, subsequent postpartum complications, and the eventual identification of adrenal insufficiency. The primary complaint was intense abdominal pain in upper and lower regions, necessitating thorough assessments via imaging and consultations with various specialties. Despite the complexity of her case, the medical team effectively managed the patient's symptoms, conducted appropriate tests, and initiated timely interventions to address postpartum complications.

The clinical relevance of adrenal hemorrhage was historically considered an autopsy finding, with an incidence of 0.14-1.8% for both unilateral and bilateral cases. Adrenal hemorrhage is a rare condition, especially before the development of advanced imaging techniques like CT and MRI [1,2,5].

Recent case studies have highlighted its occurrence during pregnancy, emphasizing its diagnostic challenges due to overlapping symptoms with other conditions like preeclampsia and placental abruption [3,4].

Other possible causes of the severe abdominal pain included bowel obstruction, pancreatitis, and urinary tract infection [6]. The interdisciplinary approach used played a pivotal role in excluding these causes and tailoring treatments. The patient's positive response to steroid therapy and pain relief emphasized the importance of considering adrenal insufficiency in cases of unexplained abdominal symptoms [1,7].

Managing the postpartum complications, ranging from anemia to fever and suspected adrenal gland bleeding, necessitated collaboration between obstetrics, hematology, and endocrinology. Spontaneous adrenal hemorrhage during pregnancy has been managed successfully with conservative approaches, including corticosteroid therapy, as described in similar cases [3,4]. The decision to administer a blood transfusion, antibiotics, and corticosteroids reflected a comprehensive strategy for managing the patient's complex condition.

This case underscores the importance of thorough clinical assessments, collaborative efforts across specialties, and timely interventions for the optimal care of pregnant patients with complicated medical conditions. The successful diagnosis and management highlight the significance of a patient-centered, interdisciplinary approach in medical practice.

## Conclusions

This case illustrates the challenges and nuances in diagnosing and managing a pregnant patient with severe abdominal pain and subsequent postpartum complications. Diagnostic imaging, laboratory tests, and interdisciplinary collaboration were instrumental in uncovering the underlying causes and guiding appropriate treatments for bilateral adrenal hemorrhage. Effective teamwork and communication among different medical teams played a crucial role in achieving a positive outcome for the patient.
